# A Server-Based Mobile Coaching System

**DOI:** 10.3390/s101210640

**Published:** 2010-11-30

**Authors:** Arnold Baca, Philipp Kornfeind, Emanuel Preuschl, Sebastian Bichler, Martin Tampier, Hristo Novatchkov

**Affiliations:** University of Vienna, Centre for Sports Science and University Sports, Department of Biomechanics, Kinesiology and Applied Computer Science, Vienna, Austria; E-Mails: philipp.kornfeind@univie.ac.at (P.K.); emanuel.preuschl@univie.ac.at (E.P.); sebastian.bichler@univie.ac.at (S.B.); martin.tampier@univie.ac.at (M.T.); hristo.novatchkov@univie.ac.at (H.N.)

**Keywords:** body sensor network, mobile device, ANT, expert feedback system

## Abstract

A prototype system for monitoring, transmitting and processing performance data in sports for the purpose of providing feedback has been developed. During training, athletes are equipped with a mobile device and wireless sensors using the ANT protocol in order to acquire biomechanical, physiological and other sports specific parameters. The measured data is buffered locally and forwarded via the Internet to a server. The server provides experts (coaches, biomechanists, sports medicine specialists *etc.*) with remote data access, analysis and (partly automated) feedback routines. In this way, experts are able to analyze the athlete’s performance and return individual feedback messages from remote locations.

## 1. Introduction

Coaches and athletes require effective methods to improve sports performance. As a consequence of advances in technology, systems have been constructed to present relevant sports specific feedback information to athletes during and shortly after training and competition. Such feedback systems are primarily designed to assist sportsmen and to monitor their training for the purpose of achieving better performance [1], but also for avoidance of excessive fatigue. In the motor learning literature there is evidence that feedback combined with practice is a potent factor for affecting motor skill learning [2]. Significant improvements of sports skill performance as a result of appropriate feedback are reported. Feedback systems are used to acquire performance data and present processed information on the motor task performed, which is not directly observable. Integrated and coordinated approaches from sports science (biomechanics, motor learning, exercise physiology, sports psychology), engineering and computer science guarantee a high level of training economy and enable the guidance and control of the training process continuously [3].

It’s not always obvious what information value can be drawn from a certain dataset. Because of the volume of data acquired and/or its high complexity, athletes might not be able to draw the right conclusions during a training session. Even coaches might experience difficulties giving appropriate feedback based on the acquired data. Consequently, experts often have to be involved in order to interpret the collected data properly. Moreover, tools may be helpful that can mine the data sources for subtle and previously unknown patterns, which might exist in the data pertaining to performance or excessive fatigue.

One important determinant in the development of today's feedback systems is certainly the continuous progress in up-to-date technologies such as sensors and mobile devices. In particular, due to the extensive and powerful functionalities as well as their miniaturization, such (rather high-tech) hardware equipment is more and more applicable not only for health monitoring (e.g., [4]) but also for the design of wearable solutions applied in sports activities (e.g., [5]).

Wireless sensors allow a convenient integration and easier usage during the data acquisition phase. At the same time interference with the athlete can be reduced considerably. Furthermore, recent sensor technologies have the advantage of low power consumption, allowing their use during long-term training sessions.

In addition, modern mobile devices provide their users with many gadgets that were rather unrealistic some time ago but are standard features nowadays. Moreover, the support of different communication tools, such as Internet-related technologies but also various sensor protocols, enable a wide range of mobile applications. For instance, such devices can now be used for the reception, storage and further transmission of sensor data. Even more, their networking abilities and their small design make them applicable at almost any place, in particular at sports training facilities.

Different feedback systems are already used in the field of leisure and competitive sports to improve the sports technique or just to avoid excessive fatigue [6–8]. For this kind of applications a non-interfering collection of biomechanical and physiological data as well as immediate response of assorted parameters to the performing athlete are crucial. In order to transmit performance relevant information such as position (e.g., [9]), reaction force (e.g., [10]) or heart rate data, wireless technologies are being applied increasingly. MarathonNET, for example, is a project that specializes on the monitoring of selected athletes (position, velocity and heart rate) and on the possibilities to personally keep records and analyze the recorded data with the help of an online service [11]. Specially adapted computer systems are used in cycling [12] to evaluate and control performance more efficiently. A wireless sensor network was developed to optimize position changes within an inhomogeneous group of cyclists using their physiological data. Collins and Anderson [13] report on a system for monitoring performance data in rowing using a PDA with inbuilt WLAN capabilities and a data acquisition card. Data from sensors mounted on the rowing boat is captured and transmitted to the laptop of the coach, who may give immediate feedback. Vales-Alonso *et al.* [14] present a prototype system comprising monitoring units for obtaining data both from the athlete and the environment and a Wireless Sensor Network (WSN) for communicating with these units. Based on the recorded data a decision machine directs the athletes’ training to fulfill specific goals. Ubiquitous solutions are also implemented in fitness training environments, motivating users with the help of mobile applications that support them with training advices [15].

Commercial systems like the Nokia® Sportstracker [16] are capable of recording the athletes’ training, thereby allowing other users to monitor their training data via the Internet. The implementation, though, processes only GPS and heart rate information and does not offer any feedback routines either. Athlosoft’s® [17] smartphone sports solution offers the capability of collecting several performance parameters (heart rate, pedaling cadence, speed, distance, ECG, GPS). This data is presented on the smartphone and can also be transmitted to a server to do further analysis via a web interface. To our knowledge there is no approach available that combines mobile data acquisition methods with centralized analysis routines and feedback functionality in the way the system proposed in this paper does.

Our main research goal is to establish a mobile coaching system that is capable of integrating the above mentioned information and communication technologies and makes use of their advantages for the purpose of providing intelligent “online-feedbacks”. This system requires a bidirectional information flow and a framework that includes prompt and suitable sensor data acquisition solutions as well as methodologies for the detection of relevant information during training. Sensors acquire the relevant performance data; the digitized signals are wirelessly transmitted to a mobile client and thereafter sent to a server. Coaches and sports scientists may thus give rapid feedback to athletes during training (‘online training sessions’) from any remote location providing Internet access. In addition, athletes may easily document their training, compare their performance to others or put it in relation to norm profiles.

One challenge is thereby to find efficient methods for the collection and the transfer of a large amount of data as it occurs during different sports activities and motion sequences. The intention is to design effective services for the convergence of mobile devices and sensor networks in terms of feature extraction, data reduction and information retrieval [18]. Another complex task is to implement and adapt intelligent algorithms based on the integration of training data as well as individual performance profile data into knowledge-based and expert systems for the automatic generation of feedback (e.g., [19]).

A system which is suitable to the needs of professional and amateur athletes coming from various sports is intended. The main focus, though, is set on popular sports like running, cycling and fitness. One basic idea is to integrate common smartphones with standardized protocols that are used by the majority of the population. Based on these intentions, a working prototype has been developed.

## 2. Conception

**Figure 1.**
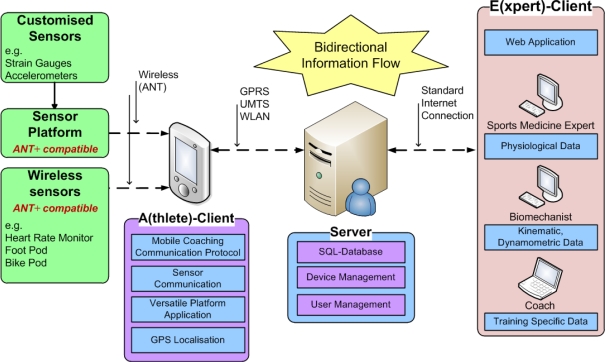
Framework of the server-based Mobile Coaching System.

In the following, a description of the system’s framework is given comprehensively by illustrating its components and the information flow. Basically, the server-based Mobile Coaching System (MCS) is an assembly of the following functional units (also shown in Figure 1):

1. Wireless sensor devices
2. Athlete-Client(s) with Internet connectivity (A-Client)
3. Web application server
4. Expert-Client(s) (E-Client)

The types of sensors used in a particular training session depend on the sports specific data that is relevant to the coach, sports scientist or athlete. Such could be physiological and biomechanical parameters but also positional data for tactical purposes. The MCS supports wireless sensors (heart rate monitor, foot pod, bike pod, bicycle power sensor *etc.*) that use the ANT+ connectivity solution (extension of the ANT protocol [20]) for communication. Moreover, by using sensor platforms with ANT+ interoperability for the acquisition of analog or digital sensor signals (e.g., from accelerometers, strain gauges) the range of supported sensor types can be broadened. The measured sensor data is transmitted to the A-Client, a mobile device (smartphone) running an application software. Such mobile devices often have integrated GPS receivers, which can be applied to obtain position and speed data of the athlete. The A-Client’s main functions are to communicate with the sensors (setup configuration, data acquisition), to pre-process the measured data and to transmit the data packets to the web application server. A graphical user interface (GUI) enables the athlete to monitor real-time sensor data and to interact with the software (e.g., to select a predefined training session). The A-Client software automatically establishes an HTTP connection and forwards the data to the server or, in case of temporary interruption, buffers the data packets locally. Depending on the signal strength of surrounding radio cells, the underlying communication interface (e.g., GPRS, EDGE, UMTS, HSDPA/HSUPA, WLAN) is automatically selected by the operating system of the mobile phone.

As shown in Figure 1, the concept is based on a centralized web application server, which is responsible for data administration, user-/device management and web-interface functionality. Via the web-interface, experts are provided with applications (E-Clients) for remote data access (in almost real time) as well as analysis and feedback routines. Feedback information is either derived directly from the measured data or by additionally considering earlier stored athletes’ training and performance data. By means of the E-Clients experts may send their advices to the athletes, who, for example, receive an alert pop-up message accompanied by a vibration or ring tone alarm on the A-Client. The MCS based on the centralized server structure thus provides bidirectional information flow. In addition, upon availability of intelligent algorithms for analysis, feedback may also be generated automatically.

## 3. Sports Specific Sensor Implementation

The complexity of a functional sensor setup in sports applications is comparatively high. Difficulties regarding the application of sensors to athletes or sport equipment need to be managed in order to minimize interferences during exercises, which are normally not performed under laboratory conditions. Important criteria are the geometric factors of a sensor (size, shape, weight), the electrical interface (wired/wireless) and additionally needed components (amplifier, power source). Obviously, the selection of inappropriate sensors can highly influence the interpretation of the data acquired. Predefined requirements for each sensor (physical range, sensitivity, accuracy, linearity, frequency behavior *etc.*) may help to prevent systematic errors and to develop a reliable sensor configuration. Built-in sensors of current smartphones enable inexperienced users to collect data easily without expensive costs. However, in many cases the accuracy and information content of the sensor data obtained is not sufficient for sports applications.

Concerning the sensor interface, an essential property of the MCS is the wireless connectivity and in particular the ANT+ compatibility to existing devices [21]. The ANT+ solution is based on predefined device profiles (e.g., heart rate monitors; speed-/distance monitors; cadence sensors) and shows the trend to become a de facto standard for sensor applications in sports. In any case, sensors from ANT+ Alliance Members (about 200+ manufacturers) can be easily connected to the A-Client using shared network keys and present one future proofing feature of the MCS.

### 3.1. ANT+ Wireless Sensor Devices

For the current implementation of the ANT interface into smartphones, a microSDIO card (SDA-323, SPECTEC, Taiwan) in combination with Windows Mobile® 6.5 is used. Because of some problems with this hardware (small range, temperature instability) the development of a universal MiniUSB-to-ANT adapter based on the Nordic nRF24AP2 radio transceiver chip (Nordic Semiconductors ASA, Trondheim, Norway) is in progress. In general, the microSDIO card as well as the Nordic chip operate at the 2.4 GHz ISM band and actually embed the ANT protocol for adaptive TDMA communication. Some outstanding features of this technology are the following:

- Ultra low power consumption (coin-cell battery life 3+ years)
- Data rate up to 1 Mbps (theoretical value)
- Efficient mechanisms to avoid message collision (up to 125 RF channels)
- Wide supported network types (peer-to-peer, star, tree, mesh)
- Transceiver and transmitter-only functionality (utile for wireless sensor nodes)

In the following sections the currently implemented ANT+ devices will be described with regard to their sensor type, measuring parameters and sampling rates. Use cases utilizing sensors in selected sports will be presented later in Section 6.

#### 3.1.1. Heart Rate Monitor

As defined in the device profile for heart rate monitors, this sensor transmits primarily the projected heart rate in beats-per-minute (bpm) as well as the time difference between two heart beats (R-R peak detection) at a sampling rate of 4 Hz. In conjunction with the heart rate, the lag can be used for calculating the heart rate variability (HRV), a subject specific parameter used for more detailed analyses (e.g., as predictor for the actual physiological load). A heart beat counter, necessary for the event detection of an R-peak (HRV calculation), is also provided by the sensor.

#### 3.1.2. Stride Based Speed and Distance Monitor (SDM)

This sensor is used to measure the number of strides taken and thus the instantaneous running speed (pace; time for 1 km) and accordingly the covered running distance can be calculated. Therefore, a prior calibration of the stride length needs to be performed by running a known distance (e.g., 800 m) or by correcting the measured distance with a simultaneously recorded GPS-based dataset. During data request it is also possible to choose between the speed & distance parameters (2 Hz, 4 Hz) or the speed and cadence parameters (2 Hz) for the transmission. Again, a counter mechanism (number of strides) is used for event detection.

#### 3.1.3. Bike Speed and Cadence Sensor

Depending on the manufacturer, these sensors can be realized as two separated devices or combined into a single sensor unit with the same functionality. The measuring of the bike speed is typically done using a magnet mounted on the wheel spokes and a sensor on the bicycle frame which senses the magnet passing. For bike cadence measurements the procedure is similar, unless the magnet is fixed on the crank of the bicycle in order to acquire the pedaling frequency. At a sampling rate of 4 Hz the sensor provides either speed (or distance), cadence or both together. Because the calculated speed (and also distance) is based on the number of wheel revolutions, it is important to know the exact circumference of the mounted wheel of the bike. Analog to the SDM, concurrently captured GPS data may be used for a calibration of the needed circumference value instead of a manual measuring. When the wheel or the crank is revolving at less than 4 Hz, it has to be considered that multiple messages may arrive describing the same event.

All above described ANT+ devices provide a unique device ID to avoid wrong pairing attempts, the energy status of the battery and a manufacturer specific data field (e.g., for product branding).

### 3.2. Sensor Platform and Customized Sensors

**Figure 2.**
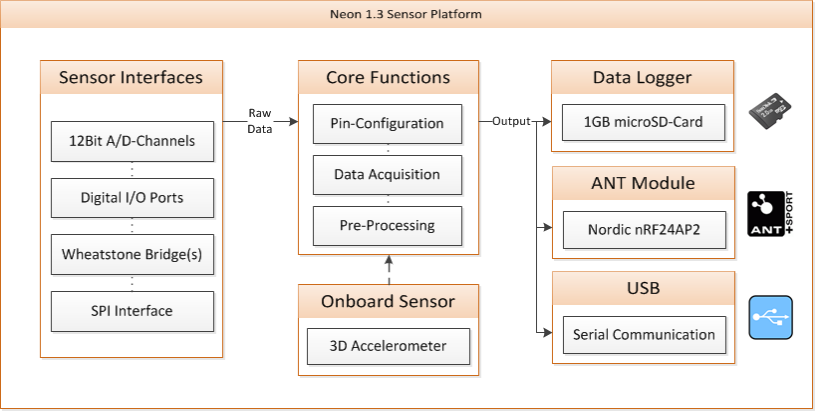
Systematic overview of the used sensor platform.

Although the number of existing ANT+ sensors is quite high, the variety is not sufficient to cover all sports when acquiring biomechanical and/or physiological parameters. Therefore, in our MCS concept we included a sensor platform [22] with the ability to integrate also non-compatible ANT+ sensors. The “Neon” platform is based on a 16-bit PIC-microcontroller and has an onboard ANT module (Nordic nRF24AP2) as well as some other useful components (for more details see Figure 2). There are several kinds of sensor input interfaces available for connecting a wide range of sensor types to the platform (analog-, digital-, and bridge sensors) and combining them to a bundled data package for transmitting it via the ANT hardware. Some adaptations in the firmware extend the platform functionality to act as an ANT+ device (new device profile, shared network keys) thus enabling an operation with the A-Client. For the acquisition of signals at high sampling rates (e.g., from accelerometers, force transducers) the integrated 1GB microSD-Card allows data logger functionality during an exercise and a transmission of the data afterwards. For configuration and/or debugging issues a USB interface may be used to remote the Neon from a computer via serial communication protocol.

In the next subsection, specific application details of some implemented customized sensors will be described (sensor type, recorded physical parameter) and aspects of required sensor specifications will be considered.

#### 3.2.1. Example: Monitoring of MTB Damping Behavior

Modern full suspension mountain bikes (MTBs) are equipped with adjustable forks and rear dampers and can be customized by varying some settings (e.g., rebound damping, travel length, preload). To evaluate the impact of these settings on the bicycle frame during cross country riding or downhill action, linear position sensors are used to acquire the dampers travel motion. In addition, accelerometers are mounted on the wheel axis and on the bicycle frame (handle bar) to obtain the resulted absorption of terrain induced shocks. Based on this information, a recommendation on the adjustment of the settings could be delivered to the biker using the MCS. Because of the fast compressions during riding, the linear position sensors need to have a very low friction in order to represent the real linear motion. Also the accelerometer needs to fulfill some minimum requirements, for instance a wide frequency range (>1 kHz), enough acceleration range (>50 g) and at least two independent axis of measuring directions. The used sensors have to offer a good resistance against dirt and water, which is of particular importance for mountain biking, a sport performed in a rough terrain.

Moreover, by applying strain gauges to selected positions on the bicycle frame, it is possible to check the mechanical stress produced by the shock vibrations during exercise. This is especially of interest when lightweight frame constructions (aluminum, carbon-fibers) are being used. Too much stress could even damage the material and lead to a broken frame. Since the Neon sensor platform supports also bridge sensors, the strain gauges can be simply combined together with the linear position sensors and the accelerometers to one wireless ANT+ sensor device connected to the A-Client.

## 4. A-Client

### 4.1. Software Concept

The mobile application running on the smartphone shall:

- Cover a wide range of sports without the need of further software updates
- Include setup procedures for the selection of trainings and authentication of athletes
- Provide methods for correct sensor assignment (e.g., when several athletes are exercising)
- Include a “real time” feedback message system
- Transmit sensor-data in “real time” to the server
- Provide reliable data transmission

In order to reach these goals, the Mobile-Coaching (MC)-Protocol has been developed to configure the ANT hardware module and to receive sensor data. Furthermore, the MC-Protocol defines the message formats used to set up the A-Client based on the athlete’s personal aims

### 4.2. A-Client Architecture

**Figure 3.**
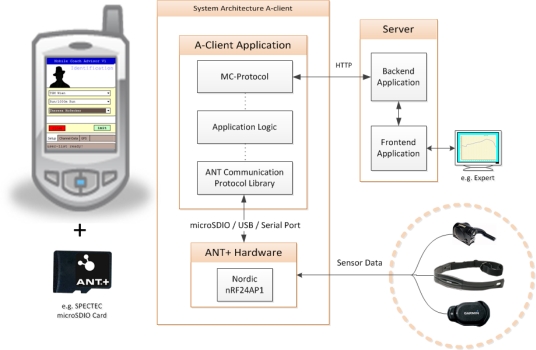
Architecture of the A-Client.

As shown in Figure 3, the implementation of the A-Client consists of three main parts. The (MC)-Protocol builds the top level of the application and is responsible for data communication with the server (MC-Backend). A more detailed description is given in the next Section (4.3).

The “Application Logic” can be seen as the connecting piece between the modules for the data transfer module and the ANT hardware. It is responsible for the control of all program processes, manages the A-Client setup and provides a GUI to the user. The lowest level includes a module that enables a direct communication with the ANT hardware. As shown in Figure 3, the connection between the A-Client application and the ANT hardware may be set up by using a microSDIO card, an USB Port, Bluetooth or a serial connection. While the MC-Protocol allows almost platform independent development (UMTS/GPRS and WLAN can be found in common smartphones), existing ANT+ compatible solutions at the moment reduce this choice to Windows Mobile® based phones and iPhones (using the iPhone ANT+ Adapter). An application porting to Android in combination with a newly developed MiniUSB-to-ANT adapter is planned for the future.

### 4.3. MC-Protocol

The developed MC-Protocol comprises nine types of message formats (see Table 1) allowing the management of training sessions and athletes. It shall be noted that it could also be used and adapted for other applications based on ANT+. All of these nine requests call the same server address via a PHP-script (mc_backend.php). The client’s action is defined by a parameter called “type”, which is sent via the request using the HTTP post method (see Section 4.5 for more details).

Depending on the request, the result is a combined string with delimiters like semicolon or asterisk (for a general description see Table 2). In case of a request fail, the output contains the keyword “error”, followed by a short error description.

The principle is illustrated by two examples.

**Example_1:**
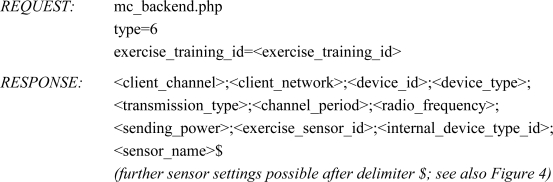
Message format #6 – sensor setup

The request of Example_1 requires the parameter <exercise_training_id>. This parameter describes a unique exercise within a training session (training sessions are split up in one or more exercises), which the athlete has chosen in the A-Client application initially (e.g., training session “Running” / Exercise “1,000 m run”). Depending on the <exercise_training_id>, the MC-Backend knows which sensors are needed for the chosen training session.

When requesting the sensor setup, the A-client can parse the received information and prepare the ANT hardware for listening to foot pods in the surrounding area. Since the sensor setup and specification are standardized in the ANT+ device profiles, there is no need to update the client software or change the MC-Protocol when integrating new sensors.

**Example_2:**
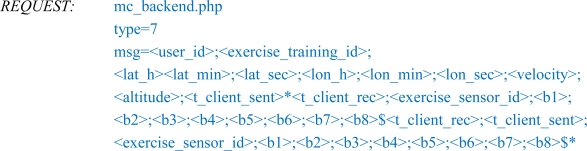
Message format #7 – sending sensor data (including actual GPS coordinates) to server

**Figure 5.**
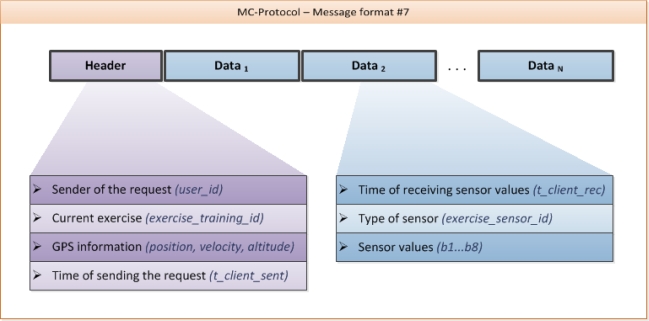
Message format #7.

As shown in Figure 5, this message format is formed by a header containing some general information and N data packets.

Since the ANT protocol defines a fixed format for the transmission of sensor data values (exactly 8 bytes), the A-Client itself does not need any detailed information about the sensors for generating the sensor data records being sent. In fact, the sensor values are evaluated in the backend, which generates a response including a feedback message.

- *RESPONSE:* <status>;<free text>

The feedback message is contained in the field <free text>. The field <status> can be used to automatically change the status of the A-Client without the need of the athlete’s interaction.

Following this strategy, there is no need for an extra message format in the MC-Protocol for receiving feedback messages, which would end up in superfluously polling the server. For instance, the response messages received from the A-Client may include the following information:

*RESPONSE 1:* 1; Please start training
*RESPONSE 2:* 2; Training stopped
*RESPONSE 3:* 3; You are too fast
*RESPONSE 4:* 4; (no new feedback messages at the moment)

As illustrated above, status 2 may automatically invoke the A-Client to stop all processes of receiving and sending sensor data and furthermore to turn back to the main setup.

### 4.4. Current Implementation of the A-Client

**Figure 6.**
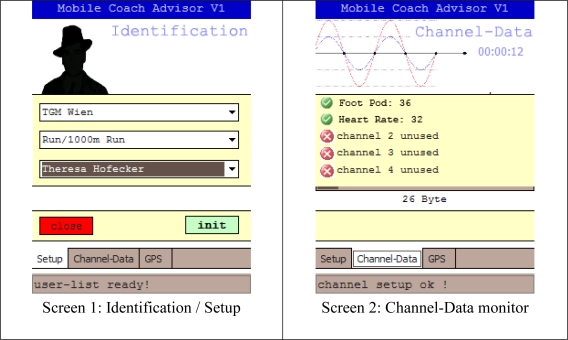

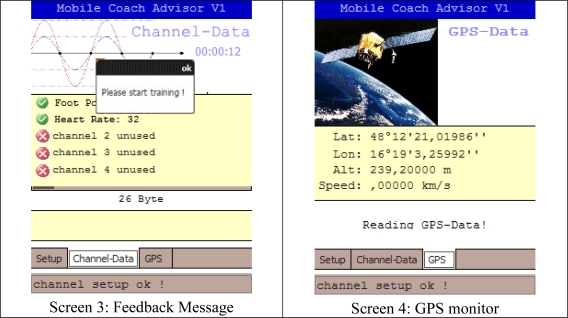
GUI of the A-Client application.

The current A-Client implementation comprises a Smartphone (HTC® Touch 2, Windows Mobile® 6.5) and the SPECTEC microSDIO card (SDA-323) for the reception of the measured signals. The mobile programs are implemented in C# using Microsoft’s® .NET Framework. As shown in the GUI design of the A-client in Figure 6 (example for 1,000 m run—Using a heart rate monitor and a foot pod as sensors), the application consists of three tabs: Setup, Channel-Data and GPS.

#### 4.4.1. Setup

Before the training session can be started, the athlete has to set up the A-Client to his/her personal aims. Corresponding to this functionality, the message formats #1–3, as described in the MC communication protocol, are used. After completing the setup, the message formats #4–6 are used to obtain the information regarding the ANT+ sensors.

#### 4.4.2. Channel-Data/GPS

After the setup, the application starts listening on different channels for incoming signals from surrounding sensors (Figure 6, top right). In addition, the current GPS location of the athlete is identified (Figure 6, bottom right). The collected data is buffered temporarily, sent to the host in defined time intervals and stored in the server’s SQL database. Furthermore, the server looks up for new feedback messages which are then automatically returned by using the response of the message format #7 of the MC-Protocol (see Table 1).

### 4.5. Data Transmission Procedure

Especially for the interpretation of biomechanical data a complete and consistent data set is essential. Therefore, the data transfer is based on the HTTP protocol with integrated handshake mechanism for sending sensor data to the server in order to provide reliable data transmission. Although UDP (typically used for multimedia streaming like videos) would provide higher transmission rates, one disadvantage is the lack of reliability. Data packets can get lost without automatic recognition of the UDP protocol itself.

However, for future developments and specific needs for the A-Client (e.g., long time measurements of sensors with high transmission rates), the MC-Protocol can be enhanced with a data packet control mechanism for establishing a reliable data transmission on the basis of UDP. In the current implementation, our focus has been set on the realization of a reliable transmission mechanism such as HTTP provides.

The data transmission is implemented by a class called “http” which provides the core mechanisms for sending and receiving data. This class contains the following methods:

- Method: void server_url (String url)
  This method enables the owner of the class-instance to set up the server URL of the backend (in our case http://www/...../mc_backend.php) at application startup.
- Method: void server_send(int message_format, string postData)
  This method enables the owner of the class-instance to send any data together with the message type information (see Chapter 4.3 MC-Protocol for example requests). It is a non-blocking command, allowing the main application to precede execution without waiting for results.
- Interface: void http_result (String result, int message_format)
  Since the method “server_send” is a non-blocking command, it is required to include an interface which is called whenever an HTTP post request (implemented in method server_send) has finished. The owner of the class-instance takes several actions depending on the message type (e.g., in case of message_format #6 – sensor setup, the A-client parses the received message to get detailed information on how the ANT+ channels have to be configured).

Theoretically, it is possible to send each sensor value in a single request in order to provide data transfer in “real time”. But considering the time delay that occurs during the establishment of a TCP connection it would end up in a very insufficient and slow data transmission which is not practicable. Therefore the definition of message format #7 allows the transfer of more than one sensor value at once. The reliable data transmission is accomplished by the use of HTTP including a simple transmission mechanism based on time triggered buffering of measured sensor values. The data is collected, put together as described in message format #7 and sent in one single request. For optimization purposes, the MC-Protocol includes the parameter for setting the “time interval of sending data to the server” (message format #4, requesting general client hardware settings).

During experiments, a practicable range for the timing interval was determined. A good value for reliable and stable data transmission without too large latency could be found for a setup of 10 seconds. However, this value can be changed without the need of recompiling the A-Client application.

**Figure 7.**
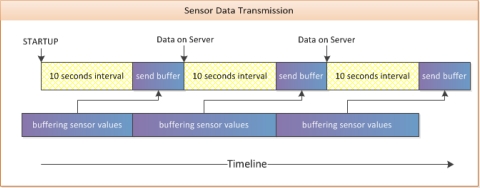
Sensor data transmission timing.

Figure 7 shows a typical timeline of sending sensor data with a fixed time interval of 10 seconds. This mechanism for timing and buffering sensor values is implemented in the main class of the A-Client application.

The transmission of sensor data is done in five steps,

1. Sensor values are buffered using a dynamically growing linear list
2. At the end of a 10 second interval, the values from the buffer are combined into a message described by the MC-Protocol (Message Format #7); the buffer is cleared afterwards
3. The method “server_send” is called to initialize the data transfer
4. The main class stops the interval timer; new incoming sensor values will be buffered again
5. The interval timer is started again as soon as the main class of the A-Client has received a response corresponding to the HTTP request

As described above and in regards to the timing shown in Figure 7, the buffered sensor values are available on the server after the preset time interval plus the time delay for sending the whole data string. In principle, it would be possible to start the interval timer again as soon as sending has begun, but this can cause some problems. For instance, when the time required for sending is higher than the selected time interval for buffering (e.g., because of a weak HTTP connection) the application would have more than one send request running at the same time.

### 4.6. Indoor and Outdoor Tests

In order to determine the quality of data transmission, several tests under lab (indoor tests) as well as rough conditions (outdoor test) have been performed. For this purpose we used a sample sensor configuration on the A-Client with established Internet connectivity to evaluate data transfer rates, delay and stability issues. During the indoor tests the mobile phones were not moved to guarantee stable Internet connection. The outdoor experiments have been done under real conditions (running trial in small forest) in order to identify disturbing effects caused by fluctuations of Internet connectivity. In Tables 3 and 4, the results of selected measurement parameters concerning the data transmission are given.

#### 4.6.1. Results of the Data Transmission Experiments

As shown in Table 3, the number of sensor values sent per second indicates large variations although the mobile phones were not moved within the whole indoor test. As a consequence, also the number of sensor values sent per interval varies remarkably. As illustrated in Table 4, the transmission mechanism is stable enough to buffer and send up to about 1,450 values within one packet. This feature is very important to compensate instable transmission rates and therefore provide reliable data transmission.

#### 4.6.2. Conclusion and Future Optimizations for Data Transmission

A stable Internet connection does not automatically guarantee constant data transmission rates. Therefore the average number of sensor values sent per second and the capability to compensate instable transmission rates are of high importance. Typical ANT+ sensors available on the market work at a message rate of 4Hz, meaning four sensors would produce 16 sensor values per second. As shown in Table 4, the current client is able to send 18 sensor values per second in rough circumstances. Therefore when assuming that a typical training includes a maximum of four sensors, the current prototype implementation of the A-Client already is applicable for sports like running, cycling or mountain biking. There are at least three possible ways on how data transmission can be optimized:

- Feature extraction for sensor data reduction on the A-Client (e.g., acceleration peaks)
- Delayed data transmission after performance (sensor data of a training session is buffered and sent afterwards)
- Using UDP for data transmission (extending the MC-Protocol with features for the establishment of a reliable data transmission)

### 4.7. Further Performance Details

The number of simultaneous users mainly depends on two factors:

- Server capabilities (most significant restriction)
  Since each A-client acts like a web browser calling a website, the capabilities of the used webserver (with focus on data transmission, number of supported simultaneous HTTP connections, write performance on database) represent the most significant restriction on the number of possible simultaneous users.
- Unique sensor identification
  ANT+ provides mechanisms to identify each sensor by using a “unique” serial number which is coded in two bytes directly on each sensor module. Since the implemented A-Client provides such a mechanism of unique sensor assignment, theoretically 65,536 users using the same type of sensor are possible.

## 5. Server and E-Client

**Figure 8.**
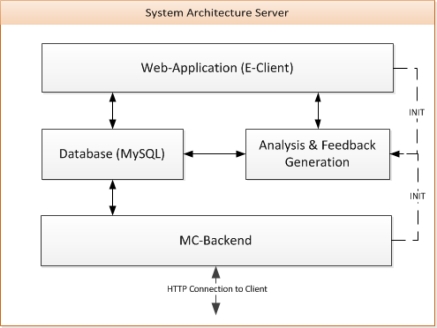
System architecture of the server.

The system architecture of the server is shown in Figure 8. MySQL was chosen as database server. The MC-Backend handles the data transfer between the A-Client(s) and the database via the MC-Protocol. This backend uses HTTP for the communication. In this way, the modules and logic for the feedback generation can be implemented in any programming language as long as HTTP and the MySQL database are supported. In our case, PHP 5 was used for all the implementations. The database can be seen as the core of the system, since the training data, the results of the data analysis and the feedback messages are stored and selected from there.

The web application (E-Client) is responsible for the configuration of the A-Client(s) as well as the user and training administration. ANT+ compatible sensors can be registered according to their specification (network key, channel id, transmission and device type, *etc.*). Sensors can be assigned to exercises, which can be combined into one training type. Depending on this type (e.g., single or group), a training session can be started by the A- or E-Client (INIT in Figure 8).

**Figure 9.**
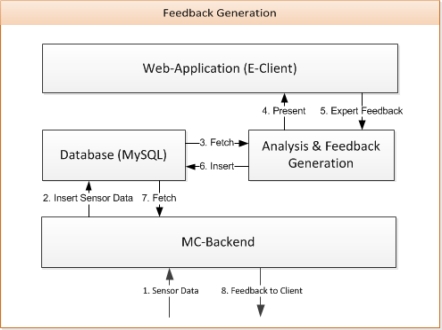
Feedback generation.

**Figure 10.**
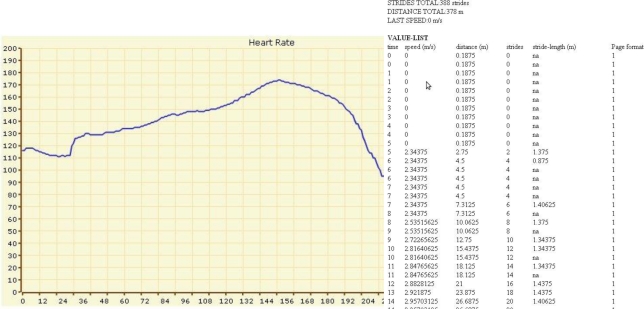
Charts and tables to present training values.

Figure 9 shows how the feedback information is generated. Once, the training is started by the A-Client, the module “Analysis & Feedback Generation” is called by the MC-Backend. Alternatively, it can be called by the E-Client via a web interface. The module runs in the background during the entire training, forwarding the training data to the web interface. In particular, experts can look at the parameterized time curves visualized by tables or charts (see Figure 10). Freely available software solutions (JQuery and OpenFlash) are used for this purpose. Experts and coaches can generate feedback based on the presented performance data, analysis results or suggestions offered by the module. The feedback is sent from the web interface into the database (Figure 9, 5. Expert Feedback and 6. Insert).

Once started, the module analyzes the training data of each athlete (in the group) while periodically checking for new data. The sub-module “Feedback Generation” makes use of, among others, knowledge-based rules of training science. In running, for example, feedback is generated by three routines: First, the current response of the load on the athlete is fetched from the database (e.g., heart rate 130 bpm). In addition, the difference to the target response (e.g., 150 bpm) is calculated. Routine 2 chooses a proper strategy to regulate and intervene in the training. For such purposes, new values of the regulation variables like stride frequency or speed are computed. One possibility would be to increase the speed gradually until the reference is reached. Routine 3 tries to facilitate the instructions and generates feedback messages, like ‘Increase your stride length, but keep the frequency’.

Currently, only simple rules like the Karvonen formula [23] are embedded in the approach. In future we intend to implement intelligent methods to assist the athlete’s training. Therefore, the extrapolation of certain performance parameters (e.g., heart rate as indicator of the physiological load) should be integrated in upcoming mathematical models. Approaches for different sports have already been reported in the literature [14,24,25]. The Rated Perceived Exertion Scale [26] could, for example, also be applied to provide individual feedback. In particular, a lot of weighted rules will be used in combination with fuzzy logic methods to calculate the regulation variables. Another goal is to individualize the feedback provision. One consideration is that athletes can customize the amount and content of the messages. The current server implementation can be accessed via a gigabit network connection, ensuring high availability.

## 6. Use Cases and Their Specific Sensor Configurations

The following use cases comprise three application fields of the MCS. Our first implementations deal with running and mountain biking, two endurance sports. Furthermore, sensors have been applied to specific exercising machines for giving feedback during resistance training. Table 5 gives a survey of sensors and parameters relevant to these sports.

In running, commercially available heart rate monitors (HRM) are used to obtain the heart rate (HR) and the heart rate variability (HRV), which represents the variation of the beat-to-beat time interval of the heart. Both parameters are good indicators for the momentary physiological load. In many sports applications standard GPS is used for the acquisition of the position and speed of an athlete. In the case of running, however, more accurate results for the instantaneous speed might be obtained using stride sensors.

HR and HRV are also of interest in cycling. In analogy to running the cadence sensor counts the number of pedalling cycles in order to calculate the pedalling frequency. The electronic gear position indicator provides valuable information for coaching novice mountain bikers, who often lack the experience in choosing the right gear to challenge a climb. Appropriate feedback would be useful for them. Speedometers provide information on instantaneous velocity of the athlete. The A-Client’s built in accelerometers can be used as inclination sensors providing information on the angle of gradient of the slope.

**Figure 11.**
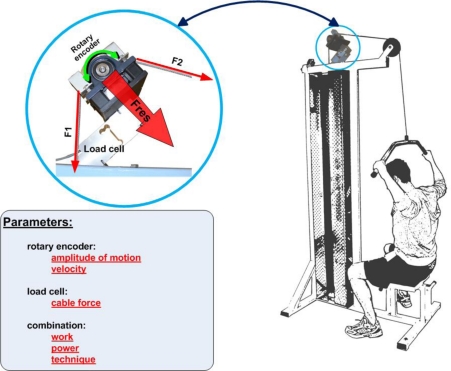
Implementation of a load cell and a rotary encoder into an exercising machine.

Training machines are widely used in order to strengthen certain muscle groups. Various variants of exercising techniques cause different loads on the musculoskeletal system. Hence, the determination of the exercise’s motion profile is essential [27]. The implementation of a force transducer (load cell) and a rotary encoder (used to measure the motion of the weight/handle bar) in a training machine is highlighted in Figure 11. This setup can be used to measure the weight’s travel distance and the applied force on the handle bar in order to estimate work and power of the exercising motion. Information on the quality of the technique like the smoothness of the motion may be obtained and appropriate feedback can be delivered.

## 7. Conclusions and Future Work

Tools and methods aiming at the improvement of athletes’ sports performance and/or avoidance of excessive fatigue are advancing continuously in the last couple of years. The miniaturized design of recent wireless sensors allows measuring different kinds of biomechanical, physiological or physical parameters during sports activities. Moreover, today’s mobile devices support manifold communication technologies for receiving and further transmission of sensor data.

Such high tech equipment brings therefore great advantages for athletes and coaches who can access and analyze data characterizing the activity just after completing the workout or even concurrently without having to leave the place of performance.

The great importance and practicality of such systems are illustrated by the proposed approach and highlighted by its novelty of combining feasible mobilizing possibilities with server-based feedback methods integrating modern information and communication technologies. One essential feature is to provide remote access for coaches and other specialists to parameter values (performance data, *etc.*) of exercises currently performed and return specific recommendations or instructions in real time. We consider this bidirectional approach as particularly innovative aspect of the proposed system. Moreover, the experts’ feedback may also be based on athletes’ performances from the past as well as their achievement potentials in order to allow the comparative analysis. The integration of online methods for data analysis including the potential automatically generated notifications based on intelligent algorithms is another major innovation of the approach.

Further work concentrates on the implementation of intelligent routines for the notification via automatically generated feedback messages. Meta-models [19] and time series analyses will, for example, be used for modeling the interdependency of load and performance in sports activity. It must, however, be conceded that this is a nontrivial task. Sports science, especially training science, does not hold simple rules, how training can be successful. Even when having a certain training goal, the regulation variable is often not known. In addition to numeric and statistical methods, fuzzy-logic, pattern recognition, modeling and simulation have proven to be promising methods to evaluate sports activities and to derive feedback information.

Recent studies [28] report about the increase of diseases (e.g., obesity) due to sedentary lifestyle and lack of motivation to do physical exercising among teenagers. Therefore, we are currently developing a mobile motion advisor system (MMA) [29], which adapts the concept of the MCS to the needs of exercise and sports in an educational environment. Its focus is set not on performance enhancement, but, much more, on the development of a healthy mental attitude towards exercising.

## Figures and Tables

**Figure 4.**
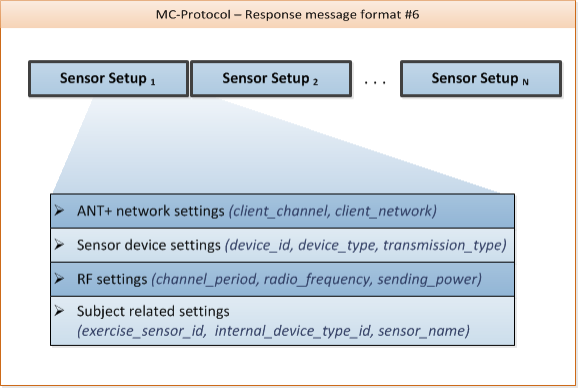
Response message format #6.

**Table 1.** Message formats used in the MC-Protocol.

#	**Function**

1	Requesting available institutions (e.g., clubs, schools)
2	Requesting current training session corresponding to an institution (including setup information about autostart and autostop of training on the A-Client)
3	Requesting members of a corresponding institution
4	Requesting general client hardware settings (e.g., device type, baud rate, GPS availability, frequency of sending data to server)
5	Requesting ANT+ network keys
6	Requesting ANT+ sensor setup (sensor name, sensor type, ANT channel, ANT network, further sensor parameters following the ANT protocol)
7	Sending sensor data to the server (plus requesting feedback messages)
8	Start / stop training
9	Pause / resume training

**Table 2.** General delimiter description.

**Description**	**Key**	**Function**

Semicolon	;	Delimiter between data values
Dollar	$	Delimiter between data records
Asterisk	*	Delimiter between different data record types

**Table 3.** Transmission results of the indoor test.

**10 second interval, 4 emulated sensors**	**Indoor Test 1**	**Indoor Test 2**

Duration	28 min	27 min
Number of sensor values	22,419	24,179
Number of intervals	105	103
kB sent (without header information)	883 kB	951 kB
Bytes per sensor value 1 (avge)	39,4 B	39,3 B
Number of sensor values sent per second (avg;max;min)	**35**; 73; 3	**38**; 66; 4
Number of sensor values sent per interval (avg;max;min)	**215**; 489; 41	**236** ; 541 ; 71
Sending time per interval (avg;max;min)	**6** s; 25 s; 2s	**6** s; 22 s; 2 s
Transmission time until storage in database (avg;max;min)	**11 s**; 35 s ; 2 s	**11 s**; 32 s; 2s

1 ASCII coded.

**Table 4.** Transmission results of the outdoor test.

**10 second interval, 3 real sensors**	**Outdoor Test 1**	**Outdoor Test 2**

Duration	78 min	18 min
Number of sensor values	39,106	8,666
Number of intervals	261	65
kB sent (without header information)	1,550 kB	341 kB
Bytes per sensor value (avg)	39,6 B	39,3 B
Number of sensor values sent per second (avg;max;min)	**18**; 60; 2	**18**; 80; 1
Number of sensor values sent per interval (avg;max;min)	**150**; 1459; 51	**134**; 441; 4
Sending time per interval (avg;max;min)	**8** s; 130 s; 2 s	**7**;56 s; 1 s
Transmission time until storage in database (avg;max;min)	**13** s; 140 s; 2 s	**12** s; 66 s; 1 s

**Table 5.** Parameter/sensor combination in selected sports.

	**Sensor**	**Parameter**
**Running**	Stride Sensor	Distance, cadence, velocity
**Running/Cycling**	Heart rate monitor (HRM)	HR, HRV
	GPS	Position, velocity
**Cycling/Mountain biking**	Gear position indicator	Used gear, distance per stride ratio
	Inclinometer	Inclination
	Cadence sensor	Pedalling frequency
	Speedometer	Speed, average speed
**Resistance training**	Dynamometer	Force
	Rotary encoder	Motion amplitude
